# Well-being at work and Finnish dairy farmers─from job demands and loneliness towards burnout

**DOI:** 10.3389/fpsyg.2022.976456

**Published:** 2022-11-01

**Authors:** Marja K. Kallioniemi, Janne Kaseva, Hanna-Riitta Kymäläinen, Jari J. Hakanen

**Affiliations:** ^1^Bioeconomy and Environment, Natural Resources Institute Finland (Luke), Jokioinen, Finland; ^2^Natural Resources and Bioproduction, Natural Resources Institute Finland (Luke), Jokioinen, Finland; ^3^Department of Agricultural Sciences, University of Helsinki, Helsinki, Finland; ^4^Work Ability and Working Careers, Finnish Institute of Occupational Health (FIOH), Helsinki, Finland

**Keywords:** agriculture, burnout, job demands – resources model, dairy farm, farmer, Finland, structural equation model, well-being

## Abstract

**Objectives:**

Novel information about the relationships between farmers’ job demands, lack of resource, burnout, and ill health is reported based on testing the so-called “health impairment process” of the Job Demands─Resources Model (JD-R) on a representative sample of Finnish dairy farmers. The aim was to find out whether two different job demand factors; workload, societal demands and lack of resource; loneliness, were related to the indicators of ill health *via* burnout.

**Methods:**

The data is based on a postal survey of 400 Finnish dairy farms. Altogether 265 questionnaires were received from 188 farms and included in the analysis. The response rate was 47 per cent among sample farms. Structural equation modelling (SEM) was used to analyze the relationships between the variables. Explanatory factor analysis was used to group the job demand and lack of resource variables.

**Results:**

We identified two job demand factors, which we labelled workload and societal demands and one lacking job resource, loneliness. Our theoretical model was supported in that two of the factors, namely workload and loneliness, were related to ill health indirectly *via* burnout. In addition, workload was directly connected with ill health. Societal job demands were not significantly related to burnout, or to ill health.

**Conclusion:**

Our results suggest that farmers could benefit from means to reduce workload, especially the physical load. This topic needs further research as the restructuring process has increased farm enterprise sizes. There is a need to develop tools and projects to alleviate loneliness among farmers. Lack of social support, high workload, ill health, and burnout among farmers may have serious direct and indirect negative consequences for the sustainability of farming.

## Introduction

High job demands and mental health problems among farmers are emerging issues. According to a literature review (Lunner Kolstrup et al., 2013), dairy farmers are placed in an occupational sector with particularly high levels of psychosocial job demands and stressors, and these challenges to well-being appear to be shared across countries and cultures. Concerning to burnout symptoms (Kallioniemi et al., 2016), almost half (45%) of Finnish dairy farmers reported the slight symptoms of burnout and almost one tenth (9%) severe symptoms. Moreover, it was shown in a large-scale study (n = 2,169) that 40 per cent of Finnish farmers considered their work to be mentally strenuous, compared with 28 per cent among the working population in general (Perkiö-Mäkelä et al., 2016).

Meanwhile, climate change, the increasing use of new technology and the continuous restructuring of the farming sector will significantly change the operating environment of agriculture in many countries and bring new challenges affecting the well-being of farmers. Moreover, new trends to modify diets, emerging societal demands related to environmental aspects and animal welfare, as well as criticism of increases in farm size and even of “industrial” farming prejudice the current discussion on agriculture. During the past decade, almost every second dairy farm has stood down the production, but despite this the milk production volume has remained about the same (Ruokatieto, 2021). Many dairy farms continuing have expanded the production capacity by building new barns and utilizing technological methods and devices. The average herd sizes have increased. Animal feeding, bedding, milking, manure removal, and animal monitoring may all be carried out by technological devices. Concurrently, concern about farm animal welfare, animal instrumentalization and industrial farming has been presented (Tuyttens et al., 2022).

The main risk factors endangering farmers’ mental health seem to be similar in many countries. Arora et al. (2020) identified finances, the weather, the workload, and poor management as risk factors for farmers in the US. Based on a literature review, Yazd et al. (2019) concluded that the most severe risk factors were financial difficulties, climate variability, poor physical health, and pesticide exposure.

Loneliness among farmers has rarely been studied as a potential threat to their well-being and health. Both Ådahl (2007) and Uthardt (2009) focused on changes related to agriculture and farming life, and loneliness among farmers emerged from their research materials. Feelings of loneliness, social isolation and increasing uncertainty were observed in Ådahl’s (2007) ethnographic study. Individuals in Finnish rural villages concealed the signs of fragmentation, and therefore they tried to maintain “the facade of a good life.” Therefore, personal problems such as distress were rarely discussed with anyone other than trusted members of the nuclear family.

The societal demands related to agriculture may be considered as challenging. The national and foreign subsidy payments have been a remarkable share of farm income during the past few years, and now and then the sensibility of these payments and northern agriculture has been questioned. Concurrently, the effects of political decisions on agriculture have increased. The farm income has been low for several years as the production costs have increased more than farm income (Official Statistics of Finland (OSF), 2018). Therefore, the liquidity and loan payment capacity of farms may be in danger (Tauriainen, 2022). Especially on dairy farms, the indebtedness has increased (Official Statistics of Finland (OSF), 2015). Agricultural intensification has elevated concern related to the environmental impacts of agriculture and animal welfare. The war in Ukraine in 2022 has brought about inflation and a rapid increase in input prices, e.g., energy (Niskanen et al., 2022) and fertilizers. Changed situation in Europe has also raised discussion about food production resilience during shocks and crises.

In many European countries, the concept of well-being at work relates to occupational safety and health, workplace health promotion, mental health, the absence of disease, and work ability. It has been assessed as a dynamic concept, which is redefined in societies (Buffet et al., 2013). Kaplan-Hallam and Bennett (2017) presented a “Domains of human well-being” framework for social-impact assessment, labelling five main domains: social, health, economic, governance and cultural.

We employ the Job Demands – Resources (JD–R) Model (Bakker and Demerouti, 2017; Lesener et al., 2019) which within the past two decades has become the most frequently used theoretical framework within which to study work characteristics and employee well-being, and their various consequences, including health. The starting point of the model is the assumption that, regardless of the type of job, psychosocial work characteristics fall into one of the two groups: job demands and job resources (Bakker and Demerouti, 2017). Job demands refer to the physical, psychological, social, and organizational aspects of a job that require sustained physical and/or psychological effort or skills and are therefore associated with certain physiological and/or psychological costs (Bakker and Demerouti, 2017). They are not necessarily inherently negative, but they may evoke strain and/or stress if high levels of effort are needed and adequate recovery is not reached (Bakker et al., 2007). Job resources, in turn, refer to the physical, psychological, social, and organizational aspects that stimulate personal growth, learning and development.

The JD-R-model was originally established to understand the antecedents of burnout (Schaufeli and Taris, 2014). Later a more comprehensive formulation of the JD-R model was introduced and tested (Schaufeli and Bakker, 2004). In that expanded model two parallel processes are hypothesized: The positive “motivational process” from job resources *via* work engagement to organizational outcomes and the negative energy draining “health impairment process” from job demands *via* burnout to ill health (Schaufeli and Bakker, 2004). In addition, (lack of) job resources are theorized and also empirically found to be associated with burnout (Schaufeli and Bakker, 2004). Several studies using the JD-R model has focused either on the motivational process or on the health impairment process (Schaufeli and Taris, 2014). In the present study, we focus on the health impairment process but also included specific aspect of job resources, namely lack of social job resource, i.e., loneliness.

According to the JD-R model every occupation may include job demands and resources that are typical or possible in all jobs, such as workload and time pressure but also specific job demands and resources that are typical of it and not necessarily in most other jobs (Bakker and Demerouti, 2017). In our study, we included workload and societal demands as job demands and one specific aspect of job resources, namely lack of social job resources, i.e., loneliness.

The nature of work among farmers may be more intensive and more difficult to detach from than in many other work sectors. Working time and free time are typically more difficult to separate from each other than among workers in general. Farmers also usually live on their workplaces (farms). These elements may restrict the possibilities to social interaction and to gain social resources, such as support and communication. In this study, we labelled loneliness as lack of social job resource, similarly to a study identifying the relationship between (lacking) social resources and work engagement among breast cancer survivors (Hakanen and Lindbohm, 2008; see also Hakanen and Roodt, 2010). To our knowledge, loneliness and one of the job demands in the present study: societal demands have not been studied in the JD-R literature. This is also the first time to test the model in a very specific occupational group, namely among the dairy farmers.

High-level and constant job demands tend to increase the risk of burnout, defined as a serious disorder that affects employee well-being and disturbs one’s relationship with one’s work (Schaufeli et al., 1996). Burnout develops during long-lasting, stressful conditions and displays three core symptoms: exhaustion, cynicism and reduced professional efficacy. Exhaustion refers to chronic fatigue and lack of energy because of overtaxing work: work fully consumes one’s emotional resources. Cynicism, described as “a distant attitude towards work in general” and to other persons, is characterized by a loss of interest, joy and meaningfulness related to work. Lack of professional efficacy has been described as “the reduced feelings of competence, successful achievement, and accomplishment both in one’s job and in the organization” (Schaufeli et al., 1996). According to the JD–R model, job demands and lacking job resources may increase the risk of burnout, which in turn, may lead to impaired health and work ability (Bakker and Demerouti, 2017; Lesener et al., 2019).

**Figure 1:**
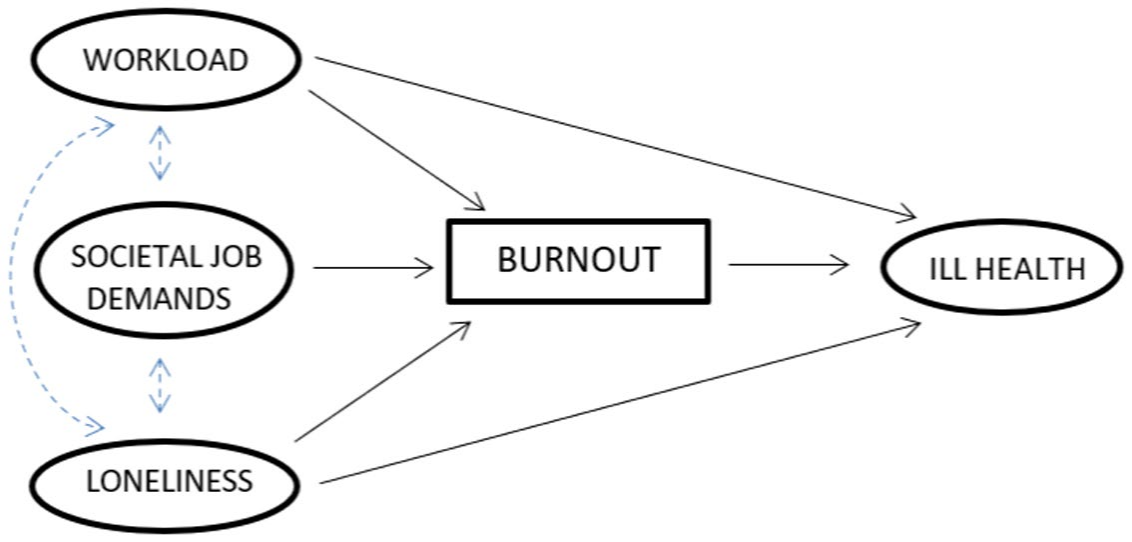
Theoretical SEM model.

We used structural equation modelling (SEM) to find out whether two different types of job demands and lack of resource were related to burnout and furthermore to ill health. The theoretical model comprising the hypothesized relationships, which are formulated at below, is depicted in Figure 1.

*Hypothesis 1*: The impact of workload in ill health is mediated via burnout.

*Hypothesis 2*: The impact of societal job demands on ill health is mediated via burnout.

*Hypothesis 3*: The impact of loneliness in ill health is mediated via burnout.

## Materials and methods

The data is based on a postal survey of 400 dairy farms, which received two postal questionnaires in two deliveries during 2010. We obtained a random sample of farms from a register kept by the Ministry of Agriculture and Forestry and targeted farmers, farming couples and persons regularly taking care of cattle (Kallioniemi et al., 2018). The cover letter included a sentence informing that the survey data will be utilized only for research purposes. Ethics approval for non-interventional study was not required.

A total of 265 completed questionnaires received from 188 dairy farms were included in the data analysis. The response rate was 47 per cent among the sample farms. Related to the JD-R model, the postal survey included questions about job resources, work engagement, job demands, burnout and the state of health. The average age of the respondents was 48 years, which was slightly lower than among all Finnish farmers (51 years) in 2010. The proportions of females and males were 44 and 56 per cent, respectively. The sampled dairy farms had an average of 29 cows and 54 field hectares (ranging between 4–220 cows and 7–365 ha, respectively); indicating the average size was larger than on average in Finland (24 cows and 37 field hectares) in 2010. The proportions of cattle barn types were nearly the same as among the farms that took part in the Finnish milk production record system. Based on the comparisons and the sample size, the sample was assessed as representative of dairy farmers (Kallioniemi et al., 2016).

The questionnaire included an instruction to assess the following items: “Assess your own experiences in your current work life. Which issues do you experience as strenuous, inconvenient, or tiring?” The respondents were asked to assess 8 job characteristics concerning workload, loneliness, and societal job demands, with a scale 1 (not strenuous at all) to 7 (very strenuous). See more detailed descriptions (Kallioniemi et al., 2016, 2018). We included two job demand factors in the model, namely “workload,” “societal job demands” and lack of resource “loneliness.” Workload included two of the demands assessed on the questionnaire, namely “physical load of work” and “amount of work”; societal job demands included “agricultural policy of the EU,” “the treatment of farmers in society and the media” and “the future of the agricultural sector.” Loneliness included “loneliness,” “lack of a companion” and “family relationships.”

Burnout was measured on the Maslach Burnout Inventory - General Survey (MBI-GS) (Schaufeli et al., 1996), which includes 16 items assessing exhaustion (5 items), cynicism (5 items) and professional efficacy (6 items). Example items; “I feel burned out from my work” (exhaustion), “I doubt the significance of my work” (cynicism) and “In my opinion, I am good at my job” (professional efficacy). The alternative responses were assessed on a seven-point rating scale from zero (never) to six (daily) (Schaufeli et al., 1996).

We assessed ill heath on four variables, including two self-assessed job demands (“own health” and “sleep difficulties”) and two questions concerning work ability. The first question related to work ability (Ilmarinen et al., 2008): “Assume that your work ability in its best has a value of 10. How many points would you give your current work ability in a scale ranging from zero to 10 (zero meaning that currently you cannot work at all)?” The second question concerned work ability up to retirement: “Do you believe that you will be able to work until retirement age given your state of health?” The response options were “no,” “probably not,” “probably,” “yes” and “I cannot answer.” In the analysis, responses to the first question were numbered (0–10) and those to the second question were assigned points: ‘no’ = 1, ‘probably not’ = 2, ‘probably =3, ‘yes’ = 4. The variable of work ability was skewed to the right, but this is usually not a problem in SEM models.”

### Statistical analysis

SEM was selected as the most appropriate method for analysing the relations between the variables, because the whole model can be tested and simultaneously take into account all the direct and indirect effects, making it possible to test all three hypotheses at the same time. First, we conducted an explanatory factor analysis (EFA) to group the demand and lack of resource variables in three meaningful factors. Kaiser-Meyer-Olkin (KMO) values greater than 0.8 indicate that the data is adequate for factor analysis. Here, the job demand factors and lack of resource factor correlated strongly with burnout, and thus the model was based on two job demand factors (workload, societal job demands) and lack of resource factor (loneliness), the structures being modified slightly during the process. Pearson correlation coefficients between the measured variables were calculated.

The Markov chain Monte Carlo method was used for the multiple imputation (MI) for missing observations on resources and demands, and the proportion of imputed observations was less than 2%. Multivariate normally distributed variables enabled the use of maximum likelihood (ML) estimation. The assumption of multivariate normality can be assessed through residuals, and both residual plots and MVN tests (Mardia’s and relative MV kurtosis) were used to establish normality. No modification indices, such as Lagrange’s multiplier test, were used to improve the model.

Goodness-of-fit of the model were evaluated by a chi-square test, which however is known to be problematic with large samples (Vandenberg, 2006). Other indices such as the Root Mean Square Error of Approximation (RMSEA) and the Standardized Root Mean Square Residual (SRMR) can be used to assess model fit, values below 0.08 usually being considered acceptable. In addition, we tested the Goodness-of-Fit Index (GFI), the Comparative Fit Index (CFI), the Normed Fit Index (NFI) and the Non-normed Fit Index (NNFI): values greater than 0.90 could be considered a reasonable fit and values over 0.95 a good fit (Hu and Bentler, 1999). We used the SAS Enterprise Guide 7.15 (SAS Institute, Inc., Cary, NC, United States) in the statistical analyses.

## Results

**Figure 2:**
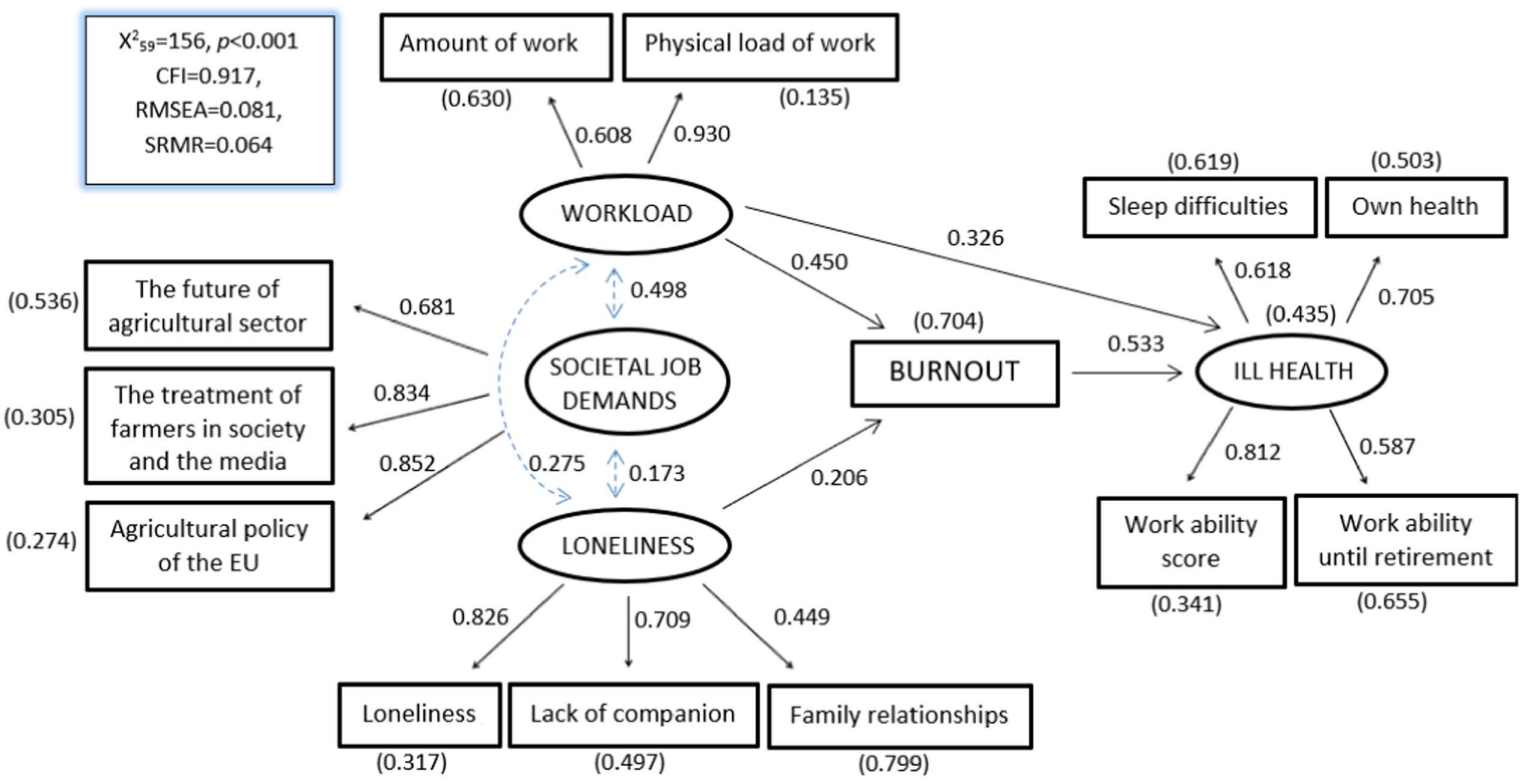
The structural equation model for burnout. Values on the double-headed arrows are correlation coefficients, those on single-headed arrows are standardized regression coefficients, and the value in parentheses is the standardized error variance. All the parameters differed from zero (*p* < 0.005), except three regression coefficients from “societal job demands” to burnout, and from “societal job demands” and “loneliness” to ill health (*p* > 0.20). Goodness-of-fit measures are shown in the box: CFI, Comparative Fit Index; RMSEA, root mean square error of approximation; SRMR, standardized root mean square residual. *N* = 250.

Figure 2 shows the results of the final hypothesized SEM model. The model fit was good or at least reasonable, according to the indices (CFI = 0.917; RMSEA = 0.081; SRMR = 0.064). The strong direct relation from the job demand factor workload to burnout (*β* = 0.450, *p* < 0.001) was explained mainly through demand “Physical load of work” (*β* = 0.930, *p* < 0.001). Lack of resource variable loneliness loaded most strongly (*β* = 0.826, *p* < 0.001) on the loneliness factor, which could lead to burnout (*β* = 0.206, *p* < 0.001). All the correlations between the factors and burnout were positive, but the relation from societal job demands was not statistically significant (β =0.057, *p* = 0.439) and was omitted from the model. Burnout had a significant association with ill health (*β* = 0.533, *p* < 0.001), which was formed from four variables related to work ability and own health. Pearson correlation coefficients between the measured variables are presented in Table 1.

**Table 1:** Pearson correlation coefficients (*r*; below the diagonal) and *p* values (H_0_: *r* = 0; above the diagonal) of the study variables (*N* = 253), grouped based on the structural equation model.

Pearson Correlation Coefficients														
Factors	Variables	1.	2.	3.	4.	5.	6.	7.	8.	9.	10.	11.	12.	13.
Workload	1. Amount of work	1	^***^	^***^	^*^	^**^	^**^	^***^	^***^	^***^	^***^	^***^	^**^	^**^
	2. Physical load of work	0.58	1	^***^	0.195	^**^	^***^	^***^	^***^	^***^	^***^	^***^	^***^	^***^
Loneliness	3. Loneliness	0.22	0.23	1	^***^	^***^	^*^	^**^	0.091	^***^	^***^	^***^	^*^	^**^
	4. Lack of companion	0.14	0.08	0.59	1	^***^	0.124	0.078	0.186	0.051	^*^	^*^	0.479	0.156
	5. Family relationships	0.20	0.17	0.33	0.39	1	^*^	^*^	0.781	^***^	^***^	^**^	0.117	0.106
Societal job demands	6. The future of the agricultural sector	0.19	0.41	0.13	0.10	0.12	1	^***^	^***^	^**^	^***^	^***^	^***^	^**^
	7. The treatment of farmers in society and the media	0.24	0.37	0.17	0.11	0.13	0.56	1	^***^	^***^	^***^	^***^	^***^	^*^
	8. Agricultural policy of the EU^a^	0.24	0.38	0.11	0.08	0.02	0.58	0.73	1	^**^	^***^	^**^	^**^	0.073
	9. Burnout	0.32	0.47	0.30	0.12	0.23	0.19	0.26	0.19	1	^***^	^***^	^***^	^***^
Ill health	10. Own health	0.37	0.46	0.24	0.13	0.25	0.25	0.26	0.26	0.40	1	^***^	^***^	^***^
	11. Sleep difficulties	0.25	0.36	0.41	0.15	0.18	0.22	0.25	0.20	0.50	0.49	1	^***^	^***^
	12. Work ability score	0.17	0.41	0.15	0.04	0.10	0.24	0.23	0.20	0.57	0.60	0.46	1	^***^
	13. Work ability until retirement	0.18	0.34	0.17	0.09	0.10	0.20	0.13	0.11	0.45	0.35	0.28	0.53	1

*** *p* < 0.001;

** *p* < 0.01;

* *p* < 0.05.

^a^EU, European union.

We used burnout as a mediator in the structural equation model, thereby facilitating the division of the effects related to a mediator into total (TE), direct (DE) and indirect (IE). Burnout partially mediated the relationships between loneliness and ill health (100% of TE, *p* = 0.004), and between workload and ill health (42% of TE, *p* < 0.001) (Table 2). Therefore, in accordance with Hypothesis 1, the impact of workload in ill health was partly mediated *via* burnout, but the impact of loneliness in ill health was totally mediated *via* burnout, in accordance with Hypothesis 3. Conversely, the results did not support Hypothesis 2, in that the third factor, societal job demands, had no statistically significant relation with either burnout or ill health. Overall, the results indicate the significance of the workload factor and its relation to ill health and burnout symptoms, which was stronger than the relation from loneliness.

**Table 2:** The total, direct and indirect effects (TE, DE, and IE) of job demand and lack of resource factors *via* burnout for ill health.

Factor	Effect	Estimate	SE	*t*-value	*p*-value
Job demand;Workload	TE	0.565	0.061	9.283	0.000
	DE	0.326	0.068	4.788	0.000
	IE	0.240	0.038	6.319	0.000
Lack of resource; Loneliness	TE	0.110	0.038	2.884	0.004
	DE	–	–	–	–
	IE	0.110	0.038	2.884	0.004

Respondent’s gender did not have statistically significant effects when added as a covariate; gender was not associated with burnout (*p* = 0.789) or with ill health (*p* = 0.387). On the other hand, when the SEM model was established separately for women and men respondents, some differences were found. The path from workload to burnout was stronger among women (*β* = 0.608 vs. *β* = 0.454), while the path from burnout to ill health was stronger among men (*β* = 0.362 vs. *β* = 0.296). Among men societal job demands were tentatively related to ill health (*β* = 0.189, *p* = 0.055), but among women this relation was not significant (*β* = −0.027, *p* = 0.777). Overall, some minor differences were observed, but respondent’s gender did not have statistically significant relationships in the SEM model.

## Discussion

We employed SEM to test the “health impairment path,” as proposed in the Job Demands─Resources Model based on survey data on 265 Finnish dairy farmers. Our aim was to find out whether the two different types of job demands and lack of resource were related to burnout and further to ill health. The health impairment process was detected in two job demand factors: workload, societal job demands and one lack of resource, loneliness. Our theoretical model was supported concerning workload and loneliness, which were indirectly related to ill health *via* burnout. Workload also had a direct relation to ill health. Societal job demands were not statistically significantly related to burnout or to ill health.

The heavy workload of farmers has been recognized in previous research. Luke (2021) found that farmers and joint farm owners worked 1.6 “full-time equivalent” (Statistics Finland, 2021) per year in 2016. Agriculture in Finland is based mainly on family farming, and people who work on dairy farms are predominantly (79%) farmers, joint farm owners, or family members. The nature of the work largely explains the amount, in that the animals must be milked and fed on weekends and holidays. Similarly, according to the European Working Conditions Survey 2015, agriculture has been mentioned among the sectors in which full-time employees work a high number of hours (on average 40.5) per week. Agriculture also scored low in quality assessments related to the physical work environment and working time (Eurofound, 2020). A possible cause for the high number of working hours may be the challenging economic situation of farms (Judd et al., 2006; European Commission, 2020; Jones et al., 2020). Corresponding to the “physical load of work” in the model, a wide survey of full-time farmers (n = 2,169) indicated that farm work is still physically heavy, and that the situation has not improved since the earlier follow-up study in 2004 (Perkiö-Mäkelä et al., 2016). In the context of European working conditions, routine physical tasks have eased in all occupational sectors except agriculture (Eurofound, 2020). Economic challenges on farms may limit the possibilities of adding to the work force and investing in work-reducing technology.

Loneliness was also significantly related to ill health *via* burnout. Many farms have ceased agricultural production in recent decades and social networks have diminished accordingly. Many farmers have to cope with their daily duties alone (Uthardt, 2009). Hansen and Østerås (2019) identified lack of social support and feelings of loneliness as stressors that may increase strain among dairy farmers. Moreover, lack of social capital may limit the transfer of knowledge, and the implementation of new technology and practices. It has also been reported in Australia (Judd et al., 2006) that isolation and loneliness were stressors that were associated with farm work and living in farming communities. The authors concluded that members of the farming community had a “limited capacity to acknowledge or express” their demands and problems, as the existing culture may dissuade individuals from expressing feelings (Judd et al., 2006). There have been other reports related to loneliness among farmers (Judd et al., 2006; Ådahl, 2007; Uthardt, 2009; Hansen and Østerås, 2019), but this topic seems to be under-researched. Our results attest to its importance given the significant relation of loneliness to ill health *via* burnout.

The third type of job demand factor included our study, societal demands (including demands; the agricultural policy of the EU, the treatment of farmers in society and the media, and the future of the agricultural sector) had no significant relation to ill health or burnout. Van den Broeck et al. (2010) applied the hindrance-challenge framework in their study and found that job hindrances were related positively to exhaustion, but negatively to vigour, and that job challenges were not related to exhaustion, but were positively related to vigour. Societal job demands in our model may represent job challenges that could be experienced as stressful, but those elevate manageable efforts, and the necessary recovery is achieved (see Bakker et al., 2007). A job demand may be considered as “a challenge or a hinderance” according to special valuation within occupation or specific context (Schaufeli and Taris, 2014; Geisler et al., 2019). Moreover, societal job demands may be rather distant for a farmer, whereas workload and loneliness are likely to be more proximal and persistent, and therefore have more negative health-related consequences.

Related to the ill health variable in the model, some literature references describe the situation among farmers. The prevalence of chronic diseases appears to be more common among farm entrepreneurs than among the working population in Finland (Perkiö-Mäkelä et al., 2016). Similarly, Beseler and Stallones (2020) found that health, the economic situation, and disease related to pesticides were key elements increasing stress among Colorado farmers. In Finland, a nationally representative population sample (n = 5,834) measured the work ability index among salaried workers, entrepreneurs with and without personnel, and farmers: the score for farmers was lower (37.9) than for the other groups and the whole sample on average (40.0) (Saarni et al., 2008). An association has been observed between the farming sector and adverse mental health outcomes such as diagnosed mental health disorders and an elevated suicide risk, according to Khan et al.’s (2019) literature review. Moreover, it is revealed in US statistics from 2016 that the proportion of suicides was the highest among workers in the agriculture, fishery, and forestry sector (Janssen, 2016). The situation in France is similar, farmers being the most likely to commit suicide among all occupational sectors and dairy farming was the most common production sector among the victims (Maeght-Lenormand, 2015). A higher suicide rate among farmers has also been reported in India (Merriott, 2016).

Farmers in distress have had access to support services related to coping therapy, economic or juridical counselling in Finland. The highest demand in 2018 was for coping therapy (Saari, 2019). Feasible methods of lighten the workload, and especially the physical load, should be available to prevent burnout and ill health. Tools and projects to facilitate loneliness are also needed. This may require a change in culture and attitudes: farmers may assess themselves as the source of the difficulty, and cultural circumstances may not encourage them to express their feelings (Judd et al., 2006). Mental health problems may thus go unnoticed. On the other hand, new innovations as well as tools such as self-assessment measures, online therapy and digital solutions simplifying social-network communication may help. These methods could compensate for the long geographical distances. Any means of improving the income level of European farmers would support the well-being of food producers and their families, including the provision of resources to improve working and environmental conditions and to facilitate the purchasing of workload-easing technological devices and methods.

Investigative journalist Nikkanen (2018) described the burnout process of a farmer in a case that included several stressors (machinery breakdowns, economic problems, heavy workload due to enlarged production, unfavourable weather conditions and lack of sleep), which in turn may strengthen the feelings of weakness, anxiety and increase alcohol consumption. All these things could lead to a negative circle whereby not all farm tasks are accomplished, farm management gradually deteriorates and working capacity is further impaired. Processes such as these are human and include elements of the same kind as in the presented model (workload, loneliness, sleeping difficulties, burnout symptoms, and lowered work ability). Although close relationships might observe some symptoms and suggest seeking help, this may not be enough. Thus, the positive, and feasible methods of intervention are needed, as Nikkanen (2018) suggests.

The strength of this study is that it is based on a representative sample of Finnish dairy farmers. However, the limitations include the cross-sectional design, and the use of self-assessment among the survey respondents. These features limit the opportunities to study causality (Van der Stede, 2014; Spector, 2019). Moreover, in that cross-sectional research is conducted over a limited time and at a certain point, the results do not give an indication of the sequence of events. On the other hand, cross-sectional studies may indicate the prevalence of outcome, risk factors and relationships among variables (Spector, 2019). The research data was collected in 2010. However, we based our study and analyses on a theoretical model that has gained wide support in a variety of different professions and sectors. Therefore, we do not think the findings to be as time-bound as, for example, studies examining the prevalence of burnout and ill health among farmers. Recent studies support the importance and timeliness of the job demands and lack of resource investigated in our study. For instance, workload has been found to be an important source of stress among Irish farm enterprises (Bondy and Cole, 2019), and a risk factor for mental health among US small farm producers (Brennan et al., 2021). In addition, as regards social relationships and loneliness, lack of support and experiences of isolation were identified as stressors among Canadian ecological farmers (Brigance et al., 2018). Moreover, Janzen et al. (2020) reported associations between diagnosed depression and lower social support among Canadian rural women and men (both farmers and non-farmers), and between depression and being non-partnered among men. Finally, more distal societal issues such as legislation and regulations have recently been reported as stressors among farmers in Georgia (Scheyett, 2022).

## Conclusion

Our theoretical model traces the process from job demands and lack of resource towards burnout and ill health, in which job demands related to the workload were associated with ill health indirectly *via* burnout. Also, lack of resource, loneliness was associated with ill health indirectly *via* burnout. Workload was also directly connected with ill health, but there were no significant relationships between societal job demands and burnout or ill health.

Lack of social support, high workload, ill health, and burnout among farmers may have serious direct and indirect negative consequences to the sustainability of farming. Farmers need practical tools and projects to mitigate their high workload and loneliness. Our study highlights the theme of loneliness among farmers, which may impair their mental health (e.g., burnout) and lead to ill health *via* lowered work ability, health problems and sleep difficulties. This theme deserves more research attention.

## Data availability statement

The datasets presented in this article are not readily available because of ethical reasons. The authors do not have the required permission to deliver information related to the state of health among our survey respondents. Requests to access the datasets should be directed to hanna-riitta.kymalainen@helsinki.fi.

## Ethics statement

The studies involving human participants were reviewed and approved by the ethical review committee of the University of Helsinki. Written informed consent for participation was not required for this study in accordance with the national legislation and the institutional requirements.

## Author contributions

MK, JK and HK participated in the research project and data collection. JK performed the statistical analysis. JH made substantial contribution to the model design and theoretical background. All authors contributed to the article and approved the submitted version.

## Funding

The research project ‘Improved well-being of dairy farmers as a means of enhancing livestock welfare’ was funded by the Ministry of Agriculture and Forestry (Makera), the Farmers’ Social Insurance Institute (Mela), and MTT Agrifood Research Finland (later Natural Resources Institute Finland, Luke).

## Conflict of interest

The authors declare that the research was conducted in the absence of any commercial or financial relationships that could be construed as a potential conflict of interest.

## Publisher’s note

All claims expressed in this article are solely those of the authors and do not necessarily represent those of their affiliated organizations, or those of the publisher, the editors and the reviewers. Any product that may be evaluated in this article, or claim that may be made by its manufacturer, is not guaranteed or endorsed by the publisher.
